# Global trends in *Cryptococcus* and its interactions with the host immune system: a bibliometric analysis

**DOI:** 10.3389/fimmu.2024.1397338

**Published:** 2024-05-07

**Authors:** Shiqin Tang, Ruiying Hao, Xin Liu, Huina He, Yanan Tian, Tingting Jing, Zhao Liu, Yanyan Xu, Xiaojing Li

**Affiliations:** ^1^ School of Clinical Medicine, The Hebei University of Engineering, Handan, Hebei, China; ^2^ Handan Stomatological Hospital, Endodontics, Handan, Hebei, China; ^3^ Department of Dermatology, Affiliated Hospital of Hebei University of Engineering, Handan, Hebei, China; ^4^ School of Clinical Medicine, The Hebei University of Engineering, Hebei Key Laboratory of Immunological Dermatology, Handan, Hebei, China

**Keywords:** *Cryptococcus* spp., cryptococcosis, host immune responses, bibliometric analysis, visualization techniques

## Abstract

**Objectives:**

This manuscript undertakes a systematic examination of the research landscape concerning global *Cryptococcus* species and their dynamism with the host immune system spanning the past decade. It furnishes a detailed survey of leading knowledge institutions and critical focal points in this area, utilizing bibliometric analysis.

**Methods:**

VOSviewer and CiteSpace software platforms were employed to systematically analyze and graphically depict the relevant literature indexed in the WoSCC database over the preceding ten years.

**Results:**

In the interval between October 1, 2013, and October 1, 2023, a corpus of 795 publications was amassed. The primary research institutions involved in this study include Duke University, the University of Minnesota, and the University of Sydney. The leading trio of nations, in terms of publication volume, comprises the United States, China, and Brazil. Among the most prolific authors are Casadevall, Arturo; Wormley, Floyd L., Jr.; and Olszewski, Michal A., with the most highly cited author being Perfect, Jr. The most esteemed journal is Mbio, while Infection and Immunity commands the highest citation frequency, and the Journal of Clinical Microbiology boasts the most significant impact factor. Present research foci encompass the intricate interactions between *Cryptococcus* pathogenesis and host immunity, alongside immune mechanisms, complications, and immunotherapies.

**Conclusion:**

This represents the first exhaustive scholarly review and bibliometric scrutiny of the evolving landscapes in *Cryptococcus* research and its interactions with the host immune system. The analyses delineated herein provide insights into prevailing research foci and trajectories, thus furnishing critical directions for subsequent inquiries in this domain.

## 1 Introduction

*Cryptococcus* is an opportunistic pathogen responsible for deep-seated fungal infections with potential fatal outcomes. It utilizes a sophisticated immune evasion strategy that frequently compromises the host organism’s immune system functionality. This yeast organism manifests in either spherical or elliptical morphologies, encased within a polysaccharide capsule. This yeast organism manifests in either spherical or elliptical morphologies, encased within a polysaccharide capsule. The capsule’s principal components consist of glucuronic acid mannose polysaccharide (GXM) and galactoxyl mannose polysaccharide (GalXM), the latter also known as glucuronic acid galactoxyl mannose polysaccharide (GXMGal) (1–3). Notably, *C.neoformans* stands as the preeminent fungal pathogen endowed with a virulent capsule, regarded as its primary virulence determinant, not exclusive to fungi but also pervasive in bacteria. Studies have demonstrated that GXM and GalXM exhibit immunomodulatory properties, thereby bolstering fungal survival through facilitation of immune evasion from the host (4). Across various biological systems, GXM and GalXM have proven efficacious in inducing cellular apoptosis. For example, investigative findings suggest that GXM within the *Cryptococcus* capsule can prompt macrophage apoptosis via mechanisms entailing the Fas and Fas-L pathways (5, 6). Moreover, Pericolini et al. reported that GalXM is capable of precipitating apoptosis in human T cells through caspase-8 activation, thereby impeding the maturation of distinct T cell responses, with the resultant adverse effect being 50-fold more potent than the suppressive action of GXM (7). Additionally, by precipitating immune dysregulation, GalXM can also promote the depletion of particular B cell populations (8). Consequently, although GXM assumes a dominant role within the polysaccharide capsule, GalXM seems to exert a more substantial regulatory influence on the host cell immune response. Such discoveries considerably enhance our comprehension of the virulence factors utilized by *Cryptococcus* in its hostile invasion of the host. Typically, this yeast is primarily transmitted via the respiratory tract, with individuals frequently becoming infected through the inhalation of airborne propagules (9).The two preeminent species, *Cryptococcus neoformans (C. neoformans)* and *Cryptococcus gattii (C. gattii)*, are renowned for their proclivity to invade human hosts. *C. neoformans* exhibits a ubiquitous distribution across the globe, whereas *C. gattii* manifests a predilection for temperate, subtropical, and tropical climes (10–12). The incursion of *Cryptococcus* represents a significant health hazard, not solely to the immunocompromised—such as individuals undergoing corticosteroid therapy, those living with HIV, and patients presenting with antifungal drug resistance—but equally to immunocompetent hosts, who might unwittingly shelter latent infections. Typically, *C. neoformans* harbors an augmented affinity for assailing immunocompromised patients, while *C. gattii* is characterized by a comparatively subdued prevalence of infection, yet retains the capacity to afflict those with intact immune defenses (13, 14). At present, the therapeutic approach to cryptococcosis is predominantly centered around staged combination pharmacotherapy, with prevailing inclinations favoring the deployment of amphotericin B concomitant with flucytosine as the principal antifungal course of action (15). However, notwithstanding the administration of these mycotic therapeutics, patient mortality rates persistently reside above the 20% threshold. In addition to the hepatorenal toxicity linked with these pharmaceuticals, there is likewise an alarming escalation in antifungal drug resistance. Consequently, the therapeutic intervention and governance of cryptococcosis continue to pose a fundamentally arduous challenge, especially in the context of immunocompromised individuals (16). Immunosuppression constitutes a pivotal element in the etiology of cryptococcosis, with immune effector cells forming the cornerstone of the host’s defense mechanism against this affliction. For those beset by *Cryptococcus* infection, it is imperative to not merely confront the malady itself, but also to fortify the host’s immune constitution.

A survey of the extant literature reveals that the intricate symbiosis between fungal entities and the host immune system has captivated significant scholarly interest. Yet, heretofore, the scholarly community has not embarked on a bibliometric scrutiny of the corpus of research concerning *Cryptococcus* and its dynamic engagement with host immune mechanisms. Consequently, this investigation harnessed the Web of Science Core Collection (WoSCC) to amass an anthology of literature concerning *Cryptococcus* and its reciprocal engagement with host immunity spanning the preceding decade. Leveraging two preeminent visualization instruments, CiteSpace and VOSviewer, the study executed both quantitative and visual scrutinies. To decipher the contemporary landscape and progressive contours of international research on the dynamic interplay between *Cryptococcus* and host immunity, this inquiry endeavors to furnish an exhaustive exegesis of the field’s evolutionary course. This encompasses pinpointing pivotal figures and ascertaining the prevailing state of research, in addition to forecasting imminent research trajectories and potentialities within this sphere.

## 2 Research methods and data sources

### 2.1 Research methods

Bibliometrics, a discipline markedly divergent from traditional narrative reviews, epitomizes a quantitative research paradigm underpinned by publication metrics, citation analyses, and textual data examination. It endeavors to delineate and elucidate the intricacies and progressive developments inherent to a scholarly discipline or research domain (17, 18). The yield of bibliometric inquiries extends beyond mere descriptive statistics to embrace the rigorous exploration of keywords, textual content, citation patterns, authorial contributions, institutional affiliations, and bibliographical references. Such explorations meticulously examine the frequency, interconnections, centrality, and clustering phenomena among authors and textual assemblages. Consequently, investigators habitually harness bibliometric methodologies to probe the evolutionary trajectories of a subject matter, discern publication inclinations, map authorial citation nexuses, and other constitutive elements (19).

VOSviewer accords primacy to the fabrication of visual representations, harnessing the potential of keywords, co-authorship dynamics, institutional collaborations, geopolitical distributions, and scholastic entities. It proffers an eclectic array of visual perspectives, encompassing Label Visualization, Density Visualization, Cluster Density Visualization, and Scatter Visualization. Within each graphical rendition, the magnitude of labels and circles serves as a visual corollary for their prominence within the designated field. The genesis of these visual depictions obviates the need for auxiliary computational tools, thereby underscoring the simplicity embodied in the mapping process and the aesthetic allure it provides (20).

CiteSpace leverages a synthesized compendium to dissect discrete modules, employing similitude algorithms to manifest graphical representations spanning a multitude of temporal dimensions. This facilitates the visualization of evolutionary trajectories and pivotal shifts within the scholarly domain (21, 22).

Although visualization tools may adeptly delineate evolutionary patterns within a scholarly arena, they might falter when it comes to apprehending the quintessential substance embedded in the literary corpus. Consequently, it is paramount to assimilate the foundational essence via conventional literary perusal techniques and to elucidate the overarching schema, progressive contour, and avant-garde vectors of the field through the aid of visualization tools. Such a methodology is indispensable for scrutinizing the evolutionary dynamics of *Cryptococcus* within the host immune system and for curating the most germane scholarly works for examination and distillation.

### 2.2 Data sources

**Figure 1:**
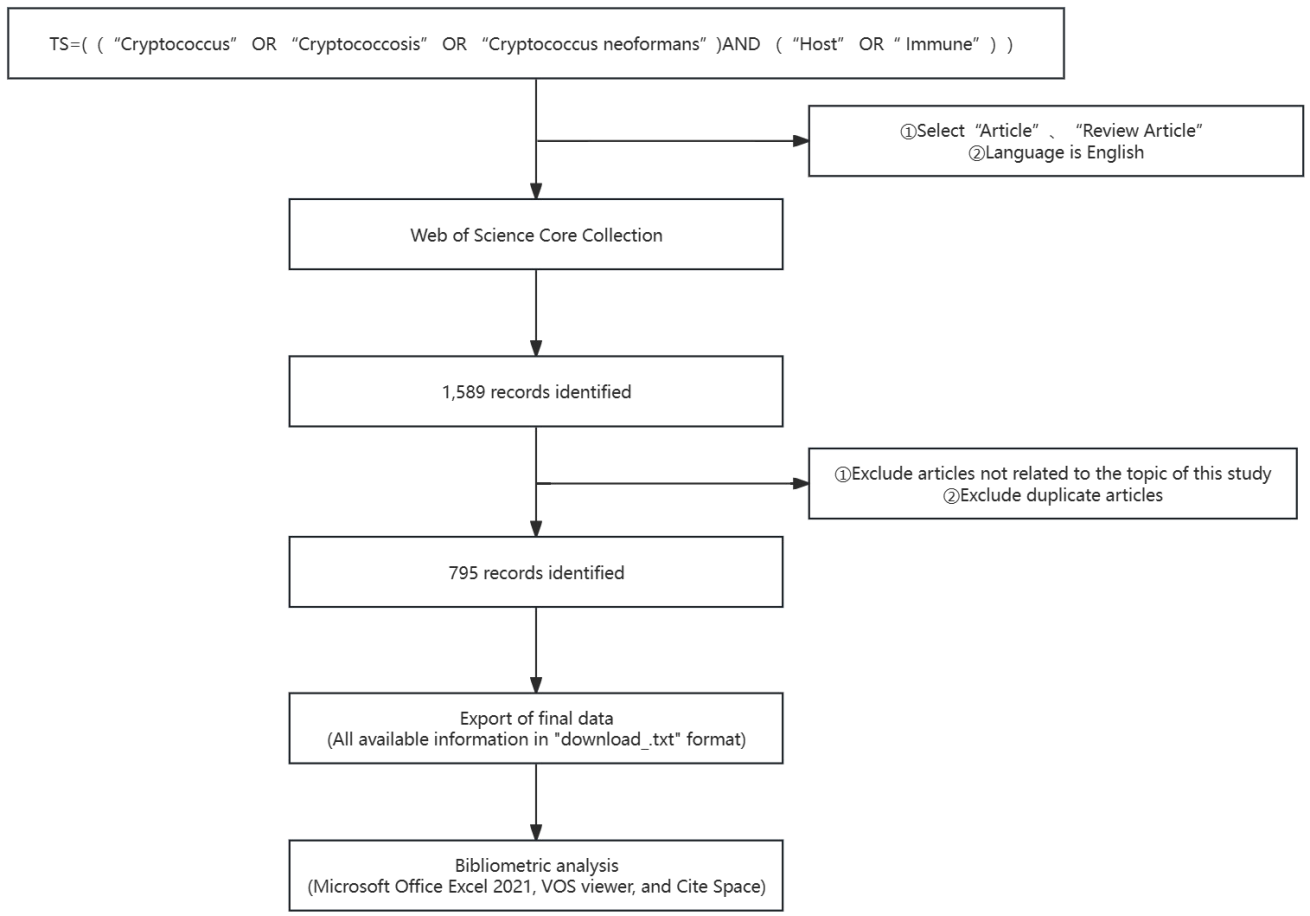
Illustrates the process of publication selection.

The systematic retrieval methodology employed within the WoSCC encompassed the following stratagem: 1. The topical search was crafted utilizing the following algorithm: TS = [(“*Cryptococcus*” OR “Cryptococcosis” OR “*Cryptococcus neoformans*”) AND (“Host” OR “Immune”)]. 2. The scholarly outputs were refined to encompass “Article” and “Review Article”, with the stipulation of English as the language of publication. 3. The temporal parameters were demarcated from October 1, 2013, through to October 1, 2023. 4. The collation of data for this inquiry was executed on October 20, 2023, a measure instituted to obviate the inclination of bias that might emanate from the database’s continual daily refreshes. Extraneous papers that did not align with the investigative theme of this study were systematically excised. The preliminary probe yielded 1589 manuscripts. Nevertheless, recognizing the propensity for redundancies and tangential materials to surface via the direct application of the search algorithm, a meticulous preprocessing was undertaken grounded on the aforementioned exclusionary criteria before any analytical endeavor. Upon rigorous scrutiny, a total of 795 valid scholarly references were distilled. The harvested bibliographic data were meticulously preserved as “full text records and references” in TXT format. The extracted corpus of literature included such elements as titles, names of authors, affiliations (inclusive of research establishments, academic institutions, hospitals), abstracts, periodical titles, dates of dissemination, and bibliographic references. Thereafter, the curated corpus of literature was meticulously transferred into a Microsoft Excel spreadsheet to facilitate subsequent analytical processes, as depicted in **Figure 1**.

## 3 Bibliometric analysis of the papers

### 3.1 Number of papers published

**Figure 2:**
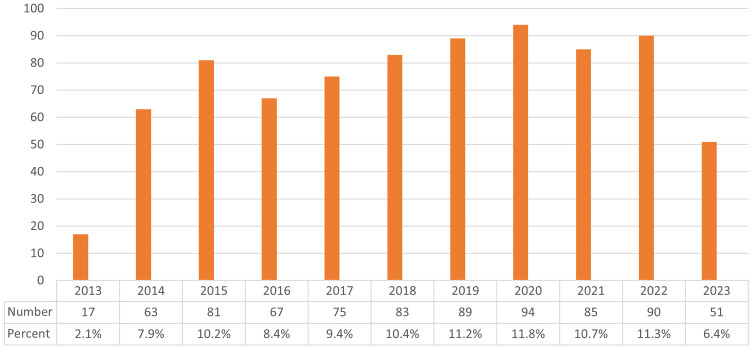
Depicts the annual publication output related to *Cryptococcus* and host immunity research from 2013 to 2023.

Fluctuations in the annual number of publications may signify shifts in research paradigms, intensity of scholarly inquiry, and evolving trends within the discipline. These 795 manuscripts originated from 3,495 authors across 63 nations and 956 institutions, featuring in 247 distinct journals and referencing 24,210 articles from 3,360 publications. **Figure 2** illustrates the chronological dissemination of scholarly works related to *Cryptococcus* and host immune interactions. Collectively, the domain experienced a notable diminution in publication frequency in 2013, accompanied by marginally lower outputs in 2014 and 2016. Nonetheless, the volume of publications consistently surpassed 70 annually throughout the period extending from 2013 to 2023, achieving a zenith in 2020 and largely preserving equilibrium subsequently.

### 3.2 Institutions and countries

**Figure 3:**
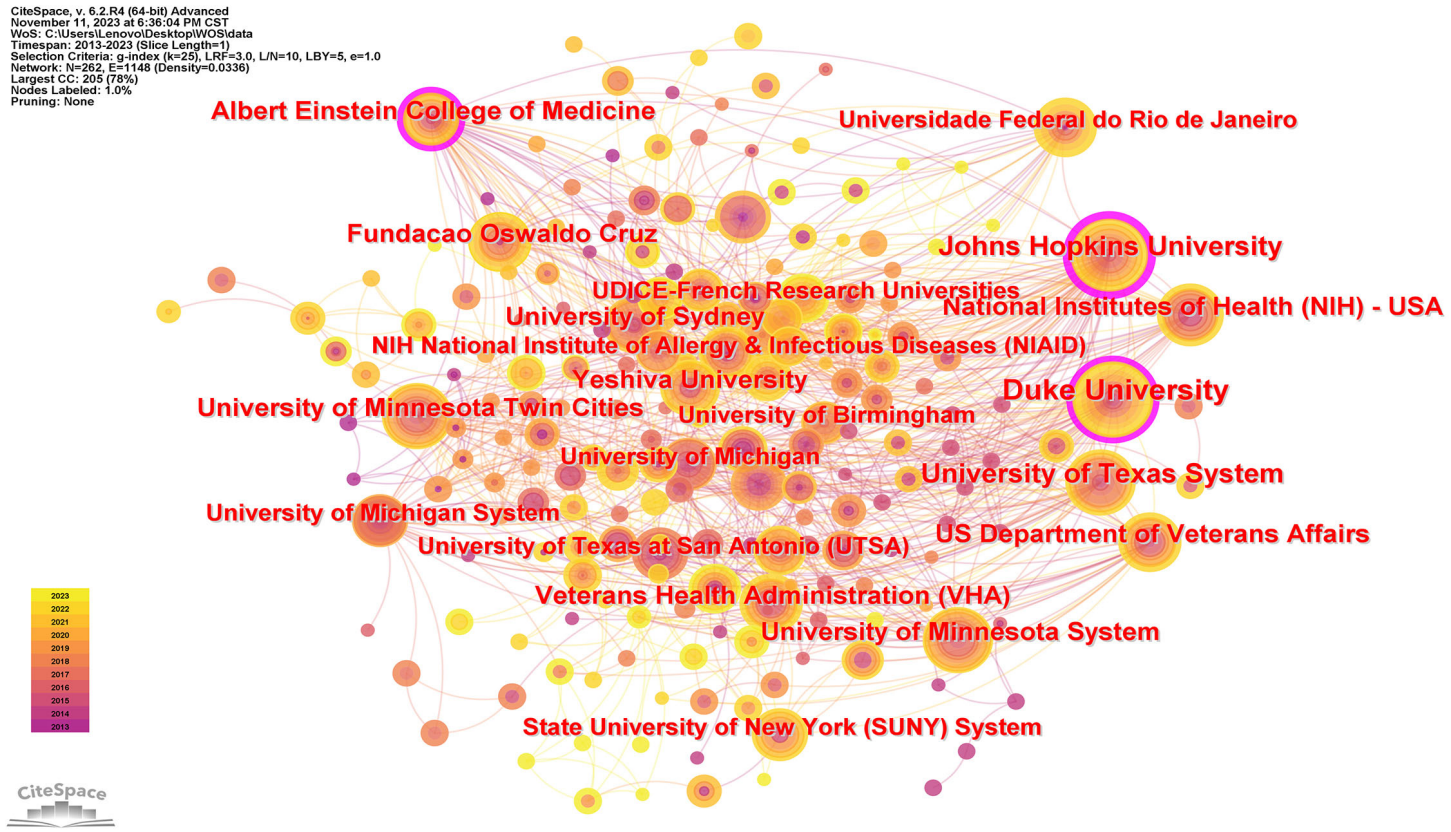
Visualization of the Institutions in the Field of *Cryptococcus* and Host Immunity Research.

**Table 1** delineates the premier 15 institutions, with Duke University at the vanguard of the global echelon through its contribution of 48 papers, succeeded by the University of Minnesota with a corpus of 33 papers, and the University of Sydney with 27 scholarly articles. It is salient to acknowledge that within the cadre of these preeminent 15 institutions, the preponderance originates from the United States (N=7), with Brazil (N=3), the United Kingdom (N=2), Australia (N=1), China (N=1), and Uganda (N=1) trailing. Following this, the formulation of a visual network illustration of institutional synergies (**Figure 3**) elucidated that Duke University sustains intimate collaborations with the University of Minnesota, Johns Hopkins University, Albert Einstein College of Medicine, University of Sydney, and Northeastern University. Furthermore, the Second Military Medical University engages in dynamic collaboration with Rutgers State University, NIAID, University of Sydney, and the Federal University of Rio de Janeiro.

**Table 1:** Top 15 countries and institutions in the field of *Cryptococcus* and host immunity research.

Rank	Country	Counts	Percent	Institution	Counts	Percent
1	USA	391	39.4%	Duke University (USA)	48	14.6%
2	China	139	14.0%	University of Minnesota (USA)	33	10.0%
3	Brazil	101	10.2%	University of Sydney (Australia)	27	8.2%
4	England	66	6.7%	Albert Einstein College of Medical (USA)	25	7.6%
5	Australia	48	4.8%	University of Texas-San Antonio (USA)	24	7.3%
6	Canada	40	4.0%	Universidade Federal do Rio de Janeiro (Brazil)	23	7.0%
7	Japan	37	3.7%	NIAID (USA)	23	7.0%
8	Germany	29	2.9%	University of Sao Paulo (Brazil)	19	5.8%
9	France	27	2.7%	University of Birmingham (UK)	19	5.8%
10	South Africa	26	2.6%	University Federal do Rio Grande do Sul (Brazil)	16	4.9%
11	Uganda	20	2.0%	Johns Hopkins Bloomberg school of public health (USA)	16	4.9%
12	India	20	2.0%	University of Washington (USA)	15	4.6%
13	South Korea	19	1.9%	Makerere University (Uganda)	15	4.6%
14	Thailand	15	1.5%	St Georges university of London (UK)	13	4.0%
15	Colombia	14	1.4%	Second Military Medical Universi (China)	13	4.0%

**Figure 4:**
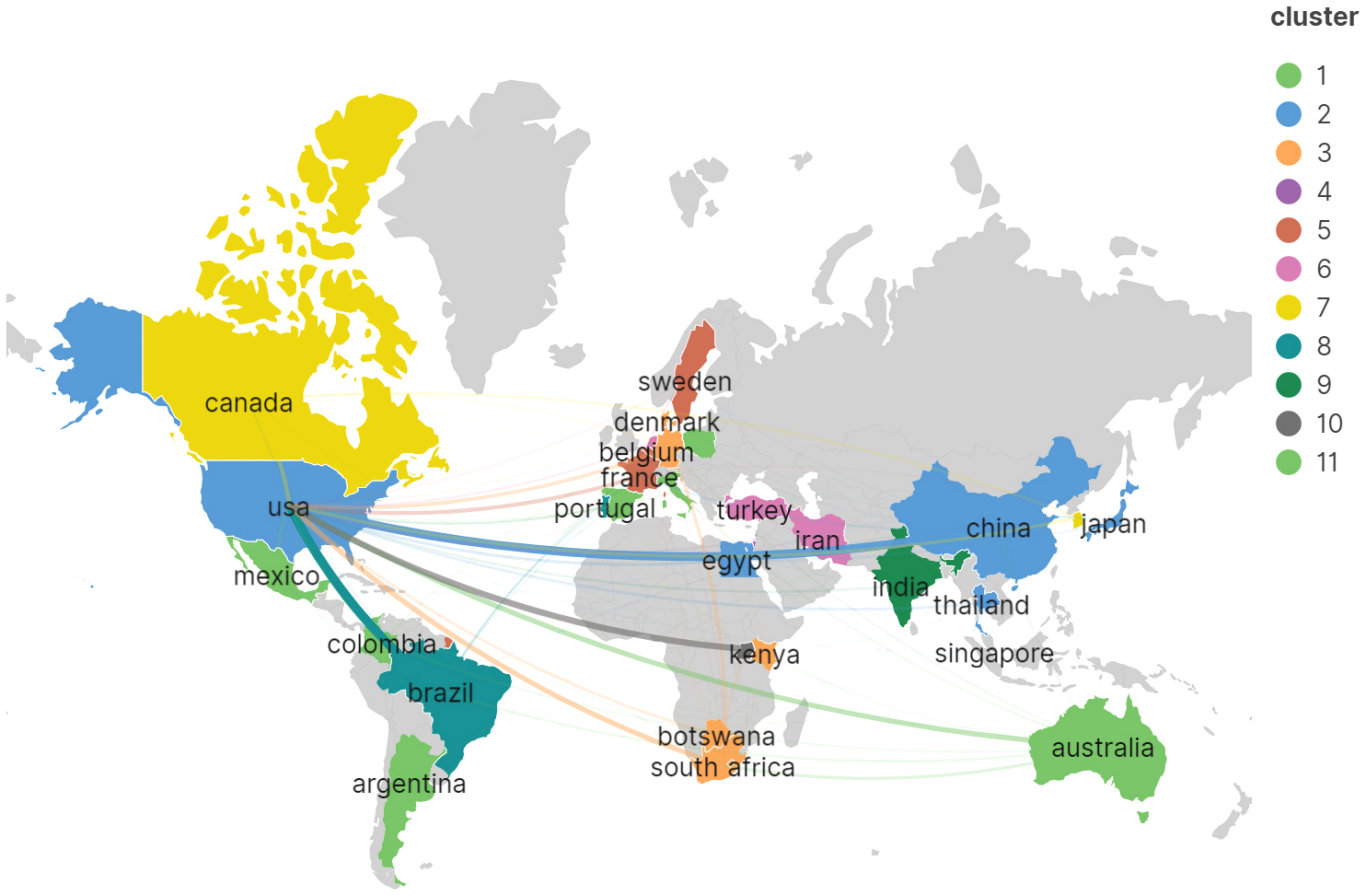
Visualization of the international network of collaboration in the research field of *Cryptococcus* and host immunity.

To delineate the nations that have made the most substantial contributions to *Cryptococcus* and host immunity research over the decade spanning 2013–2023, this investigation analyzed the scholarly output of 63 countries. As demonstrated in **Table 1**, the United States stands at the forefront with a total of 391 scholarly works, amassing 9,867 citations with an average citation rate of 25.24, markedly outstripping China, which has produced 139 scholarly works yielding 1,695 citations at an average citation rate of 18.80, and Brazil, with 101 scholarly works accompanied by 1,894 citations at an average citation rate of 12.20. Subsequently, a graphical representation was created for countries with a minimum of three publications (refer to **Figure 4**), wherein the robustness of the interconnections between nodes was delineated by the links’ thickness, signifying augmented collaboration in scholarly works among the respective nations. The chromatic differentiation of the nodes denoted distinct clusters. It is manifest that the distribution of nations contributing to this field’s literature is highly disparate, with a pronounced ‘top effect’ wherein the majority of scholarly works are penned by academics from a select consortium of nations.

### 3.3 Author analysis

**Figure 5:**
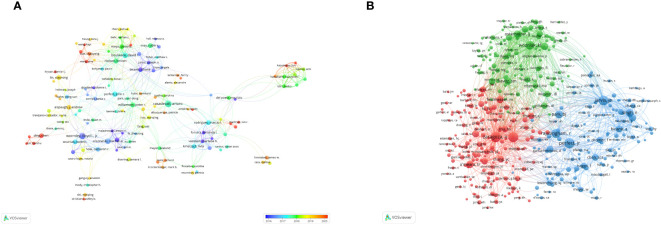
Visualization of authors in *Cryptococcus* and host immune research from 2013 to 2023 **(A)**. Visualization of co-cited authors in *Cryptococcus* and host immune research from 2013 to 2023 **(B)**.

The frequency of citations garnered by academic papers stands as a quintessential barometer of their scholarly impact. Among the globally prolific scholars, a cadre of 3,495 researchers has rendered contributions to the corpus of knowledge concerning *Cryptococcus* and host immunity. Notably, each member of the distinguished coterie of the top 10 authors has disseminated in excess of 10 scholarly papers within this domain. Amongst these authors, a quartet has each amassed in excess of 300 citations. Most noteworthy are Perfect Jr. (N=347), Zaragoza, O. (N=336), Casadevall, A. (N=330), and Jarvis, J. N. (N=317), who spearhead the citation tally (refer to **Table 2**). Predicated on these data, we devised an intricate network graph of collaborations (see **Figure 5A**) for those authors who have disseminated a minimum of five scholarly works. A complement of 141 authors satisfied this criterion. Authors were segregated into distinct consortiums, with temporal dynamics accentuated via a chromatic coding schema. Each vertex within the graphical representation signifies an individual scholar, with the magnitude of the circle mirroring their scholarly output. The interlinking lines signify collaborative incidences amongst divergent authors. Casadevall, Arturo, Wormley, Floyd L. Jr., Olszewski, Michal A., Boulware, David R., and Perfect, John R. are denoted by the most prominent vertices within the graph, in recognition of their preeminent publication count in the pertinent discipline. For example, scholars such as Casadevall, Arturo, Perfect, John R., Williamson, Peter R., Liao, Wanqing, et al., exhibit a tightly interwoven collaborative matrix. Subsequent scrutiny divulged that upon imposing a minimum citation echelon of 20 for data filtration, 391 auteurs surmounted this benchmark, culminating in the genesis of a co-citation network graph (**Figure 5B**) predicated upon this dataset. For instance, a marked synergy is observed amongst a spectrum of co-cited luminaries, including Perfect, Jr., Rajasingham, R., Jarvis, Jn., and Park, bj.

**Table 2:** Top 10 authors and co-cited authors in *Cryptococcus* and host immune research from 2013 to 2023.

Rank	Authors	Count	Co-Cited Authors	Citations
1	Casadevall,arturo	29	Perfect, jr	347
2	Wormley, Floyd l.,Jr.	25	Zaragoza, o	336
3	Olszewski, michal a.	21	Casadevall, a	330
4	Boulware, david r.	18	Jarvis, jn	317
5	Perfect, john r.	17	Rajasingham, r	264
6	May, robin c.	16	Wozniak, kl	247
7	Nielsen, kirsten	16	Singh, n	201
8	Williamson, peter r.	16	Park, bj	198
9	Lin, xiaorong	15	Huffnagle, gb	181
10	Meya, david b.	15	O'meara, tr	179

### 3.4 Journal analysis

Commencing in 2014, there has been a consistent augmentation in scholarly articles addressing *Cryptococcus* and host immunity, signifying an escalating academic intrigue in this domain. This upward trajectory is projected to persist, with a sustained volume of publications anticipated through the culmination of 2023. These treatises have been disseminated across 247 disparate journals, with the foremost 15 periodicals enumerated in **Table 3**. The periodical MBIO (N=52, 11%) has amassed a total of 1482 citations, with an average citation rate per article of 28.5, thereby securing its position at the apex. In close succession are the Journal of Fungi (N=46, 9.7%), Frontiers in Immunology (N=31, 6.6%), Infection and Immunity (N=27, 5.7%), and Frontiers in Cellular and Infection Microbiology (N=25, 5.3%). The journal with the most consequential impact factor is Nature Communications (IF=16.6).

**Table 3:** Top 15 journals in the field of research related to *Cryptococcus* and host immune interactions.

Rank	Journal	IF	Q	Publications	IF	Citations	Average Citation / Publication
1	Mbio	6.4	Q1	52	6.4	1482	28.5
2	Journal of Fungi	4.7	Q2	46	4.7	725	15.8
3	Frontiers in Immunology	7.3	Q2	31	7.3	513	16.5
4	Infection and Immunity	3.1	Q2	27	3.1	596	22.1
5	Frontiers in Cellular and Infection Microbiology	5.7	Q2	25	5.7	319	12.8
6	Journal of Immunology	4.4	Q2	22	4.4	591	26.9
7	Plos One	3.7	Q3	19	3.7	287	15.1
8	Scientific Reports	4.6	Q3	19	4.6	397	20.9
9	Medical Mycology	2.9	Q3	18	2.9	223	12.4
10	Plos Pathogens	6.7	Q1	15	6.7	424	28.3
11	Frontiers in Microbiology	5.2	Q2	14	5.2	573	40.9
12	Fungal Genetics and Biology	3	Q3	13	3	440	33.8
13	Nature Communications	16.6	Q1	13	16.6	581	44.7
14	Mycoses	4.9	Q2	13	4.9	129	9.9
15	Open Forum Infectious Diseases	4.2	Q3	13	4.2	151	11.6

As delineated in **Table 4**, it becomes manifest that within the cadre of the top 15 co-cited journals, a septenary has each amassed in excess of 1000 citations. Infection and Immunity (Co-Citations=4036) reigns supreme, succeeded by the Journal of Immunology (Co-Citations=2170), Clinical Infectious Diseases (Co-Citations=1890), Plos Pathogens (Co-Citations=1655), and Plos One (Co-Citations=1554). The periodical wielding the most distinguished impact factor is the Journal of Clinical Microbiology (IF=36.8).

**Table 4:** Top 15 journals cited in the field of research related to *Cryptococcus* and host immune interactions.

Rank	Co-cited Journal	IF	Q	Co-Citations
1	Infection and Immunity	3.1	Q2	4036
2	Journal of Immunology	4.4	Q2	2170
3	Clinical Infectious Diseases	11.8	Q1	1890
4	Plos Pathog	6.7	Q1	1655
5	Plos One	3.7	Q3	1554
6	MBIO	6.4	Q1	1483
7	The Journal of Infectious Diseases	6.4	Q2	1009
8	Medical Mycology	2.9	Q3	899
9	Proceedings of the National Academy of Sciences of the United States of America	11.1	Q1	757
10	Journal of Biological Chemistry	4.8	Q2	684
11	Molecular Microbiology	3.6	Q2	651
12	Antimicrobial Agents and Chemotherapy	4.9	Q2	607
13	AIDS	3.8	Q2	602
14	Journal of Clinical Microbiology	36.8	Q1	560
15	Cellular Microbiology	3.4	Q2	535

**Figure 6:**
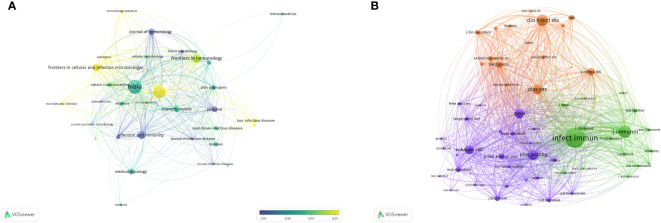
Visualization of Journal Publications in the Field of Research Related to *Cryptococcus* and Host Immune Interactions **(A)** and Visualization of Co-cited Journals **(B)**.

**Figure 7:**
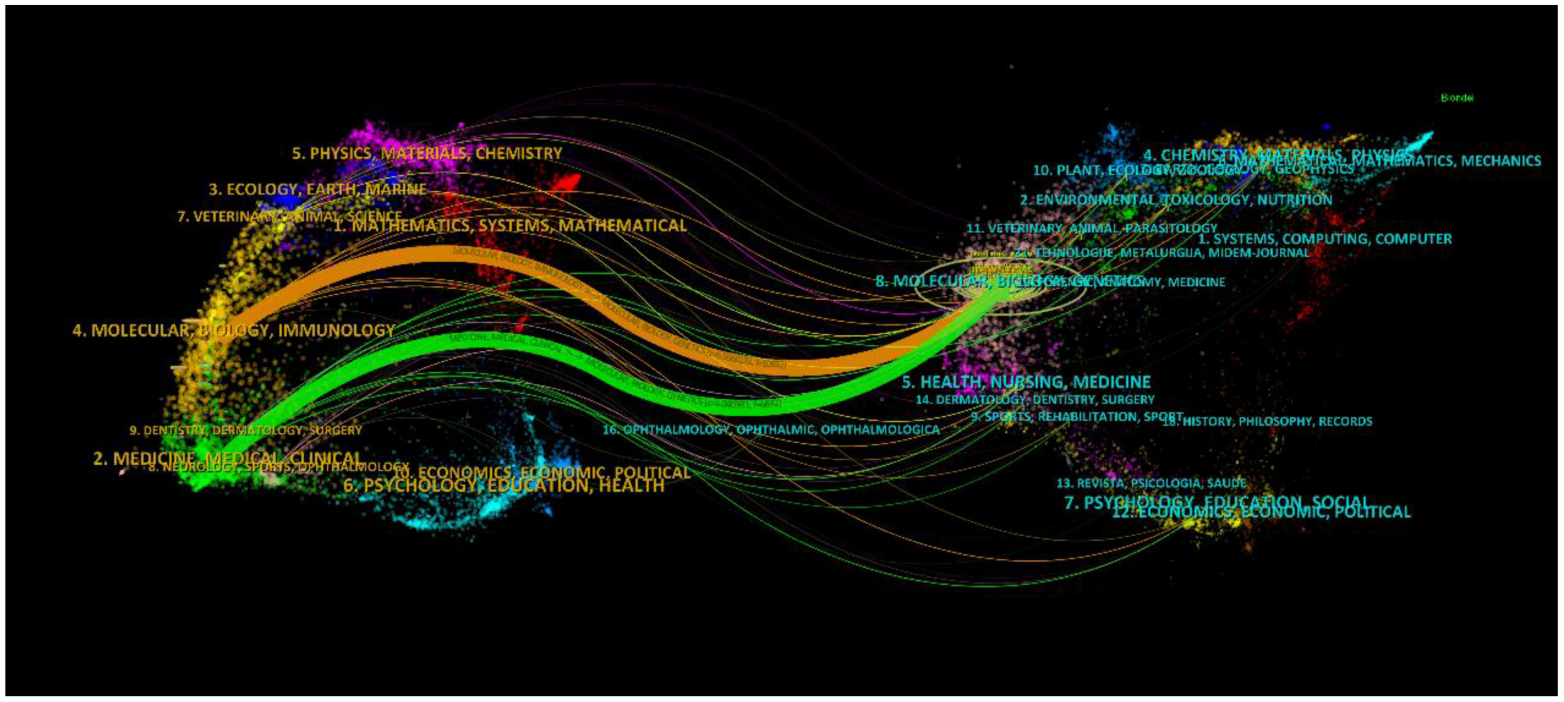
The superimposition of two graphs representing journals related to research on *Cryptococcus* and host immune interactions.

An aggregate of 33 scholarly periodicals was discerned via VOSviewer, each boasting a minimum publication frequency of five scholarly articles, culminating in the construction of a journal network diagram (**Figure 6A**). It is noteworthy that dynamic citation interconnections emerged among such journals as Mbio, Journal of Fungi, and Frontiers in Immunology. Subsequently, a filtration criterion predicated on the minimal co-citation count (encompassing 154 journals) was instituted, resulting in the curation of 60 journals for the construction of a co-citation network diagram (**Figure 6B**). The bi-directional mapping overlay of periodicals elucidates the symbiotic relationships between the citing and co-citing entities, with the left delineating the citing periodicals and the right those being co-cited. As depicted in **Figure 7**, the orange trajectory denotes the primary citation conduit, signifying that research promulgated in the journal Molecular Biology Genetics is principally cited by treatises within the journal Molecular Biology Immunology. The verdant citation trajectory intimates that research emanating from Molecular Biology Genetics is habitually cited by the journal Medicine Medical Clinical.

### 3.5 Co-cited references and references bursts

**Figure 8:**
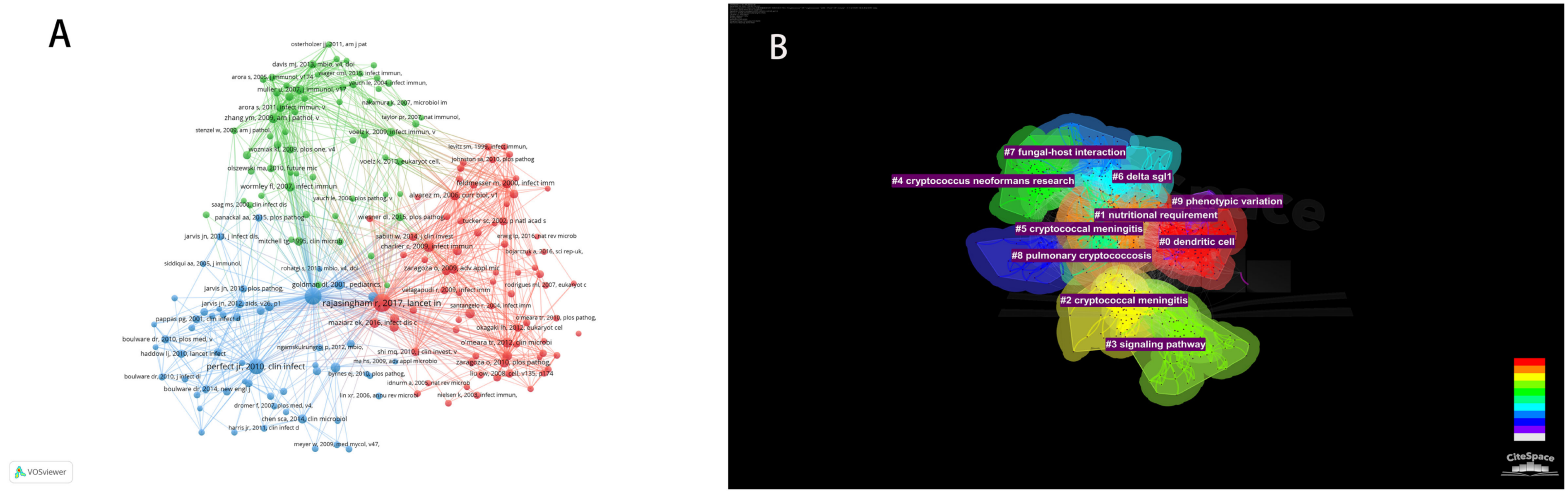
Visualization **(A)** of co-cited references on *Cryptococcus* and immunology research. Cluster network diagram **(B)** of co-cited references.

In the decade spanning from October 1, 2013, to October 1, 2023, a total of 24,210 citations were interchanged pertaining to scholarly references that explore the interplay between *Cryptococcus* and host immune responses. Among the most prominent 10 co-cited works (**Table 5**), each reference received no fewer than 60 co-citations, with a triumvirate of these works being co-cited in excess of 150 instances. Thereafter, works garnering 25 or greater co-citations were meticulously selected to fabricate the co-citation network diagram (**Figure 8A**), wherein the magnitude of the circles is proportionate to the citation frequency, thereby reflecting the scholarly significance of the works. For example, vigorous co-citation dynamics were observed among such notable works as “Rajasingham R, 2017, Lancet Infect Dis,” “Perfect JR, 2010, Clin Infect Dis,” and “Maziarz EK, 2016, Infect Dis Clin N Am.” **Figure 8B** delineates ten discrete clusters elucidated via CiteSpace, encompassing: dendritic cell dynamics, nutritional imperatives, cryptococcal meningitis, intracellular signaling cascades, *C. neoformans* investigations, delta sgl1 gene influence, fungal-host interplay, pulmonary cryptococcosis, and phenotypic plasticity.

**Table 5:** The top 10 co-cited references on *Cryptococcus* and immunology research.

Rank	Co-cited reference	Citations
1	rajasingham r, 2017, lancet infect dis, v17, p873	217
2	park bj, 2009, aids, v23, p525	197
3	perfect jr, 2010, clin infect dis, v50, p291	167
4	charlier c, 2009, infect immun, v77, p120	85
5	zaragoza o, 2009, adv appl microbiol, v68, p133	77
6	o'meara tr, 2012, clin microbiol rev, v25, p387	74
7	eldmesser m, 2000, infect immun, v68, p4225	74
8	alvarez m, 2006, curr biol, v16, p2161	70
9	kwon-chung kj, 2014, csh perspect med, v4,p123	68
10	zaragoza o, 2010, plos pathog, v6,p232	67

**Figure 9:**
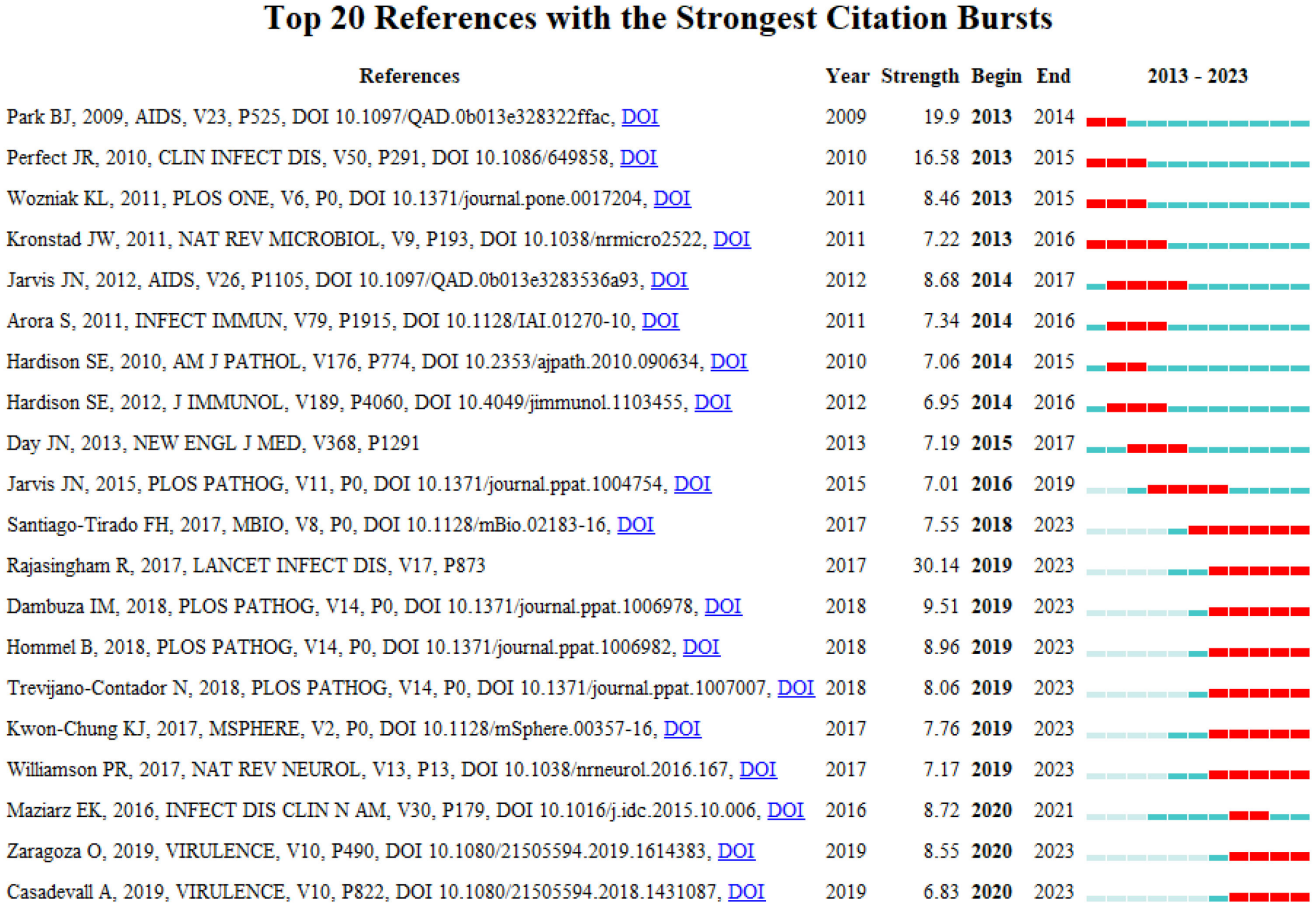
The top 20 most frequently cited references.

A burst citation denotes a reference that is frequently cited by scholars in a particular field within a specific timeframe. When a set of articles is repeatedly cited, it gives rise to the formation of a conceptual cluster (23). In the present study, CiteSpace has identified 20 references exhibiting strong burst citations. As depicted in **Figure 9**, the references are arranged according to burst sequence by their initial publication years, with each bar representing a year. The red lines signify a sudden burst of high-citation references in a particular year. The work entitled “Global burden of disease of HIV-associated cryptococcal meningitis: an updated analysis”, penned by Radha Rajasingham et al (24),exhibits the most pronounced burst citation (intensity=30.14) and was sustained from 2019 to 2023. The study with the second-highest burst citation rate (intensity=19.9), authored by Benjamin J. Park et al. and appearing in AIDS (25), spanned from 2013 to 2014. Notable, as depicted in **Table 6**, the predominantly co-cited references both address the global burden of cryptococcal meningitis attributable to host immune deficiency-related diseases. The guideline ranking third in burst rate (strength=16.58), titled “Clinical practice guidelines for the management of cryptococcal disease: 2010 update by the infectious diseases society of America,” crafted by John R. Perfect et al. and featured in “Clinical Infectious Diseases,” underwent a burst from 2013 to 2015 (26).This reference focuses primarily on the enhancement of treatment strategies for cryptococcosis, addressing factors such as host immunity, site of infection, antifungal drug toxicity, and underlying conditions, with the objective of updating efficacious management guidelines and amplifying patient diagnosis and treatment outcomes. Drawing upon these findings, one may deduce that the burst strength of these 20 references spans from 6.83 to 30.14, with durations extending from 2 to 6 years.

**Table 6:** The primary research themes of the 15 cited references.

Rank	Strength	Title	Journal	Author	Year
1	19.9	Estimation of the current global burden of cryptococcal meningitis among persons living with HIV/AIDS.	AIDS	Benjamin J Park	2009
2	16.58	Clinical practice guidelines for the management of cryptococcal disease: 2010 update by the infectious diseases society of america.	Clin Infect Dis	John R Perfect	2010
3	8.46	Role of IL-17A on resolution of pulmonary C. neoformans infection.	PLoS One	Karen L Wozniak	2011
4	7.22	Expanding fungal pathogenesis: Cryptococcus breaks out of the opportunistic box.	Nat Rew Microbiol	James W Kronstad	2011
5	8.68	Adjunctive interferon-γ immunotherapy for the treatment of HIV-associated cryptococcal meningitis: a randomized controlled trial.	AIDS	Joseph N Jarvis	2012
6	7.34	Effect of cytokine interplay on macrophage polarization during chronic pulmonary infection with Cryptococcus neoformans.	Infect Immun	Shikha Arora	2011
7	7.19	Combination antifungal therapy for cryptococcal meningitis.	N Engl J Med	Jeremy N Day	2013
8	7.55	Trojan Horse Transit Contributes to Blood-Brain Barrier Crossing of a Eukaryotic Pathogen.	mBio	Felipe H Santiago-Tirado	2017
9	30.14	Global burden of disease of HIV-associated cryptococcal meningitis: an updated analysis.	Lancet Infect Dis	Radha Rajasingham	2017
10	9.51	The Cryptococcus neoformans Titan cell is an inducible and regulated morphotype underlying pathogenesis.	PLoS Pathog	Lvy M Dambuzza	2018
11	8.96	Titan cells formation in Cryptococcus neoformans is finely tuned by environmental conditions and modulated by positive and negative genetic regulators.	PLoS Pathog	Benjamin Hommel	2018
12	8.06	Cryptococcus neoformans can form titan-like cells in vitro in response to multiple signals.	PLoS Pathog	Nuria Trevijano-Contador	2018
13	7.76	The Case for Adopting the "Species Complex" Nomenclature for the Etiologic Agents of Cryptococcosis.	mSphere	Kyung J Kwon-Chung	2017
14	8.72	Cryptococcosis.	Infect Dis Clin North Am	Eileen K Maziarz	2016
15	8.55	Basic principles of the virulence of Cryptococcus.	Virulence	Oscar Zaragoza	2019

### 3.6 Hotspots and frontiers

**Figure 10:**
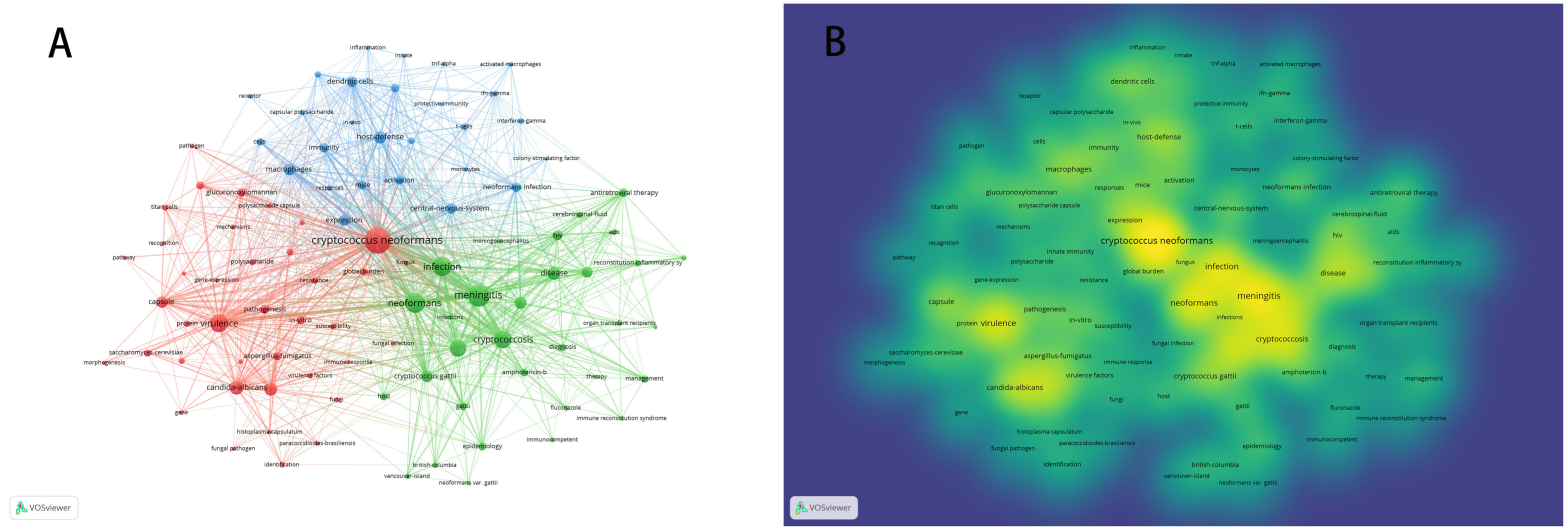
Illustrates the visualization of keyword clustering **(A)** and keyword density **(B)** in the context of research on *Cryptococcus* and host immunity.

**Figure 11:**
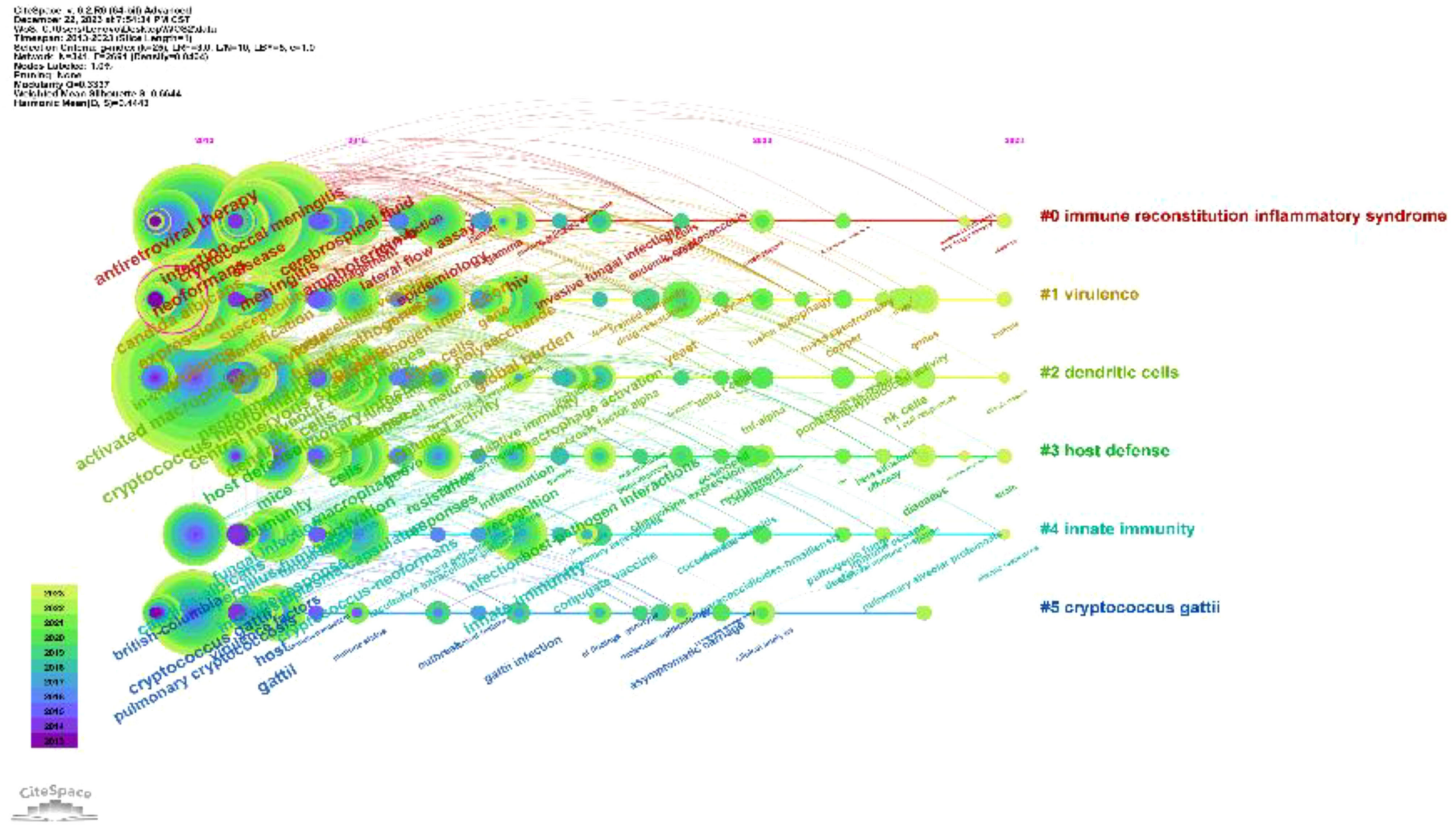
Depicts a timeline of keywords in the field of *Cryptococcus* and immune responses.

Keywords distill the quintessence of scholarly works, providing a portal through which the scholarly corpus may be navigated. A critical analysis of keywords within a specific discipline can reveal the salient themes and prospective trajectories of interest within that domain. Of the 3,083 keywords identified, 98 exceed the delineated threshold upon employing VOSviewer with a stipulated minimum keyword occurrence of 15. The aggregation and computation of aggregate connectivity strength for these 98 keywords culminate in the graphical representation of keyword clusters (**Figure 10A**) and a corresponding density visualization (**Figure 10B**). Informed by the graphical depiction of keyword networks, three pronounced clusters have materialized, each symbolizing a distinct vector of inquiry: namely, the azure cluster (delving into the host immune defense mechanisms post *Cryptococcus* infection), the crimson cluster (probing into the pathogenesis, virulence, and immune evasion strategies of *Cryptococcus* infection), and the verdant cluster (examining the immunotherapeutic interventions and recuperation from *Cryptococcus* infection). This synthesis of findings is succinctly encapsulated in **Table 7**. The magnitude of the circles within the graph mirrors the prominence of the keywords, with more substantial circles denoting augmented significance, whilst the intricacy of the linkages between nodes signifies the prevalence of keyword co-occurrences. Through the scrutiny of the timeline graph, one may perceive the fluid progression of research focal points as denoted by the keywords spanning 2013 to 2023. This chronological dissection elucidates the ascent and wane of seminal keywords, mirroring the oscillations of scholarly pursuits within the discipline. Keywords of a homogenous cluster are arrayed along a horizontal trajectory, sequenced in temporal succession from left to right, signifying the continuum from historical to contemporary. The proliferation of keywords within a collective underscores the developmental magnitude and import of the cluster’s contribution to scholarly advancements in the domain. Employing CiteSpace for keyword scrutiny, we devised a timeline graph to visually articulate the metamorphosis of research epicenters concerning the interplay between *Cryptococcus* and host immunity over the decade. The appraisal of the clustering delineation was executed via Modularity Q and Mean Silhouette metrics weighted for significance. The Silhouette coefficient, designated as S, functions as a metric for gauging homogeneity within a given cluster. An elevated S value is indicative of enhanced congruity amongst the cluster modules. A Q value in excess of 0.3 intimates a notable delineation of structure, whereas an S value surpassing 0.5 denotes a cogent clustering (27). In consonance with these thresholds, the keyword clustering module in this exposition exhibits a Q value of 0.3337 (>0.3) and an S value of 0.6644 (>0.5), thereby revealing a coherent clustering with a definitive architecture. **Figure 11** exhibits a sextet of clusters, to wit: immune reconstitution inflammatory syndrome, virulence, dendritic cells, host defense, innate immunity, and *C.gattii*. Each cluster embodies a discrete consortium, designated as #0, #1, et cetera, with the more voluminous clusters subsuming an augmented congregation of members (28).

**Table 7:** Illustrates the three major clusters of keywords to *Cryptococcus* and host immune responses.

Cluster	Color	Key words
1	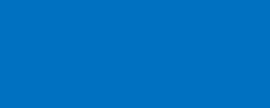	Activated macrophages、activation、alveolar macrophages、capsular polysaccharide、cells、central-nervous-system、colony-stimulating factor、dendritic cells、expression、host-defense、IFN-gamma、immune-response、immunity、in-vivo、inflammation、innate、interferon-gamma、macrophages、mice、monocytes、neoformans infection、protective immunity、Pulmonary infection、receptor、responses、t-cells、TNF-α
2	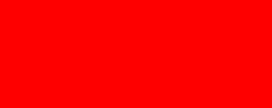	aspergillus-fumigatus、candida-albicans、capsule、cryptococcus neoformans、cryptococcus-neoformans、extracellular vesicles、fungal infection、fungal pathogen、fungal pathogenesis、fungi、fungus、gene、gene-expression、global burden、glucuronoxylomannan、histoplasma-capsulatum、identification、immune response、in-vitro、innate immunity、mechanisms、melanin、morphogenesis、paracoccidioides-brasiliensis、 pathogen、pathogenesis、pathway、phagocytosis、polysaccharide 、polysaccharide capsule、protein、recognition、resistance、saccharomyces-cerevisiae、susceptibility、titan cells、virulence、virulence factors、yeast
3	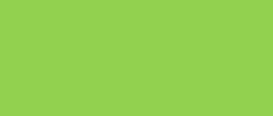	Aids、amphotericin-b、antiretroviral therapy、british-columbia、cerebrospinal-fluid、cryptococcal meningitis、cryptococcosis、cryptococcus、cryptococcus gattii、diagnosis、disease、epidemiology、fluconazole、gattii、hiv、host、immune reconstitution inflammatory syndrome、immune reconstitution syndrome、immunocompetent、infection、infections、lateral flow assay、management、meningitis、meningoencephalitis、neoformans、neoformans var. gattii、organ transplant recipients、pulmonary cryptococcosis、reconstitution inflammatory syndrome、therapy、vancouver-island

## 4 Discussion

### 4.1 General information

The corpus of literature was harvested from the Web of Science, with subsequent visual dissection of the publications undertaken via CiteSpace and VOSviewer. Ensuingly, the scholarly findings incorporated an examination of publication trajectories, authorial contributions, institutional affiliations, terminological foci, geographic provenance, periodical distributions, and bibliographic interconnections. In 2013, the domain of inquiry was in its embryonic phase. Yet, in the period extending from 2014 to 2022, there ensued a marked augmentation in scholarly outputs, with the annual production uniformly surpassing 60 treatises. The zenith of publication frequency was attained in 2020, and it is forecasted that the volume of treatises will persist in its ascension in the years succeeding 2023.

In the landscape of contemporary scientific inquiry, the host immune response has surfaced as a pivotal element in the study of cryptococcal pathogenesis. A synthesis of data from a multitude of nations and academic bodies delineates the United States, China, and Brazil as the principal nations propelling investigation in this sphere, nurturing intimate collaborative networks. Collectively, inter-nation collaboration manifests robustly across the preponderance of countries, conversely, a select assemblage finds itself within embryonic stages of scholarly pursuit, exhibiting a tempered zeal for joint scholarly ventures. A modicum of cooperation harbors the potential to propel the maturation of this investigative field and surmount scholarly impediments with heightened efficacy. Duke University (USA, N=48) distinguishes itself as the preeminent institution in publication frequency, indicative of its seminal and significant engagement within this realm of research. The hierarchical registry of periodicals manifests that MBIO (N=52), Journal of Fungi (N=46), and Frontiers in Immunology (N=31) stand as paragons of publication output in this academic field, with Infection and Immunity (Co-Citations=4,036) holding the distinction of being the most assiduously cited journal within the collective citation nexus. Additionally, the Journal of Clinical Microbiology lays claim to the most eminent impact factor, standing at 36.8.

Arturo Casadevall, of the Johns Hopkins Bloomberg School of Public Health in the United States, has ascended as the most distinguished author within this research territory (N=29). In collaboration with Floyd L. Wormley Jr., their scholarly pursuits encompass the intricacies of host immune defense mechanisms amidst cryptococcal infection, the nuances of host-pathogen interplay with *Cryptococcus*, the nature of inflammatory responses, the complexity of virulence determinants, and the pursuit of anti-cryptococcal therapeutic strategies. Their research endeavors penetrate the elucidation of underlying mechanisms at molecular, cellular, tissue, and organ levels of damage, thereby augmenting the comprehensive grasp of cryptococcal pathogenesis (29, 30). They hypothesize that in the incipient phase of cryptococcal infection, host-activated macrophages excrete extracellular vesicles (EVs) that act as pivotal ‘priming’ signals, prompting the polarization of naïve macrophages toward a pro-inflammatory phenotype and potentiating macrophage microbicidal prowess. Exploiting this salient macrophage characteristic harbors potential for the genesis of innovative immunotherapeutic modalities (31, 32). The preponderance of the top 10 references concentrates on facets of host immunity, inflammatory processes, virulence determinants, and therapeutic interventions. This focal point arguably emanates from the predilection of cryptococcal infection toward individuals with compromised immune systems, with its insidious infiltration of the blood-brain barrier culminating in cryptococcal meningitis. Furthermore, this mycotic adversary not only exhibits adaptability to the host milieu but also masterminds immune subversion, persistently engineering virulence factors to besiege the host (33–35).Hence, it remains of utmost significance to refine the modulation of the host’s immune defenses as a strategy for the immunotherapy of those at heightened vulnerability.

### 4.2 Hotspots and frontiers

Examination of high-frequency keywords can illuminate the research dynamics and emerging trends in a particular field of study. Based on keyword clustering, three principal domains have been identified to ascertain the distribution and trajectory of hotspots in the research area of *Cryptococcus* and host immunity.

#### 4.2.1 The pathogenesis of *Cryptococcus* infection

The scarlet module delineates the pathogenesis of *Cryptococcus* infection. *Cryptococcus* infection constitutes a widespread, invasive fungal infection with a global distribution, whereby virulence factors are produced during the course of infection, permeating the host and ultimately wreaking havoc on the human host (15). Contemporary investigations have elucidated that common pathogenic factors of *Cryptococcus* include adaptation to the host environment, immune evasion, and virulence factors (36). It is broadly recognized that the virulence factors of *Cryptococcus* are crucial in pathogenesis, including polysaccharide capsule, melanin, cell wall integrity, and temperature-dependent variations within the host. Nonetheless, in subsequent years, novel virulence factors have captured the attention of researchers, such as the atypical titan cells, which are deemed the optimal cellular manifestation of *Cryptococcus*, eliciting deleterious adaptive immune responses in the host and enhancing the pathogen’s survivability within the host (37–39). Similar to other fungi, *Cryptococcus* possesses the capacity to adapt and proliferate profusely under 37°C conditions, thereby precipitating the activation of pertinent signaling pathways. Strategic targeting of these virulence factors’ key loci may provide an avenue for the inhibition of *Cryptococcus* in subsequent treatments.

#### 4.2.2 The interaction between *Cryptococcus* infection and host immunity

The schematics penetrate the labyrinthine interplay between *Cryptococcus* and host immunity. Customarily, subsequent to invasion, *Cryptococcus* assumes a yeast form and enters a dormancy within the lungs of immunocompromised individuals. At the onset, the host’s immune mechanisms erect a defense against these fungi; however, should their virulence exceed the host’s tolerance, it can precipitate latent infection and even propagate to the central nervous system, resulting in fatal outcomes (40). Following invasion, the host’s initial barricade against the pathogen *Cryptococcus* is constituted by phagocytic cells, including dendritic cells, neutrophils, and alveolar macrophages (41). *Cryptococcus* possesses the capacity to orchestrate the host’s immune response to suppress inflammation, thereby circumventing phagocytic clearance and securing access to the central nervous system. Fascinatingly, research has disclosed that even subsumed by phagocytic cells, *Cryptococcus* can thrive prodigiously within these cells, and even prompt host cell lysis (42–44).Cytokines constitute diminutive molecules that facilitate interactions between various types of immune cells, frequently assuming a pivotal role in the defense against *Cryptococcus* infection. The eradication of *Cryptococcus* infection necessitates a Th1 immune response, and these protective cytokines can elicit the host Th1 immune response and amplify its efficacy. For instance, IFN-γ, TNF-α, IL-2, and IL-12 each fulfill a guardian role in forestalling *Cryptococcus* infection (45, 46). The precocious activation of TNF-α assists in the activation of dendritic cells through the classical pathway, sustaining an equilibrium of Th1/Th2 cytokines during *Cryptococcus* infection and thus diminishing host impairment (47, 48). In summation, comprehensive insight into the symbiosis between *Cryptococcus* infection and host immunity may yield advantages in circumventing immune evasion and the propagation mechanisms of *Cryptococcus*.

#### 4.2.3 Complications of *Cryptococcus* infection

The verdant module encapsulates the intricate morbidities associated with *Cryptococcus* infection. *Cryptococcus* infection primarily presents within the pulmonary system and may thereafter diffuse to the central nervous system, with the gravest consequence entailing the onset of cryptococcal meningitis. It represents the most common etiology of adult meningitis and a significant cause of mortality, particularly among individuals living with HIV/AIDS. The quantitative depletion and functional impairment of CD4+ T lymphocytes in the context of HIV infection predispose individuals to severe immunosuppression, rendering them incapable of effectively clearing *Cryptococcus* infection. This underscores the pivotal role of T cells in orchestrating host-mediated immune responses. The effective management of this disease has become a focal point of interest. Antiretroviral therapy (ART) has emerged as a potent strategy for restoring cellular immunity in individuals afflicted with HIV/AIDS. Following the administration of ART, although there is a rapid restoration of cellular immunity in individuals with HIV/AIDS-associated *Cryptococcus* infection, there is a propensity for the emergence of immune reconstitution inflammatory syndrome (IRIS). This phenomenon is characterized by a pronounced inflammatory response within the central nervous system, correlated with a heightened mortality rate among affected individuals (49, 50). Cryptococcal IRIS induces an aberrant inflammatory cascade that eventuates in host-mediated neuropathology (51). Experimental *in vivo* and *in vitro* inquiries posited that the pathogenesis of this affliction may originate from the hyperactivation of CD4+ T cells, engendering a cellular immune response that precipitates the proliferation of a myriad of inflammatory mediators within the central nervous system, including TNF-α, IFN-γ, and IL-6 (52, 53). Furthermore, the established therapeutic protocol for *Cryptococcus* infection comprises a triad of amphotericin B, flucytosine, and fluconazole. However, the pervasive incidence of complications in individuals with *Cryptococcus* infection renders the conventional treatment modalities suboptimal. Consequently, substantial scientific scrutiny has been directed towards immunotherapeutic interventions to augment the immune function of afflicted individuals, especially those deficient in CD4+ T cells. Investigations have highlighted the critical function of CD4+ T cell-mediated Th1 immune responses in forestalling cryptococcosis within animal models. For instance, the exogenous administration of cytokine IFN-γ concurrent with *Cryptococcus* therapy has demonstrated efficacy in augmenting fungal clearance from the cerebrospinal fluid (54, 55). In summation, the dynamic interaction between *Cryptococcus* and the host immune responses modulates the clinical course of *Cryptococcus* infection diseases. This necessitates an enhanced comprehension of the pathogen-host immune interface and the identification of more effective immunotherapeutic strategies to combat *Cryptococcus* infection.

### 4.3 Limitation

To guarantee the integrity of the bibliometric analysis, this investigation selected the WoSCC as the source for literature procurement. Nevertheless, owing to the rigorous standards and conventions prescribed by bibliometric analysis tools for statistical data, the study confined its data collection exclusively to journal articles indexed within the WoSCC. Despite endeavors to ameliorate this limitation, the study intrinsically grapples with potential omissions of articles within the database, which could engender a partial analysis of the data. Moreover, the quantitative dissection of data intrinsically incorporates subjective elements. Additionally, the temporal correlation with citation metrics suggests that newly published articles may accrue fewer citations relative to their predecessors, thereby rendering bibliometric indicators insufficient for assessing the merit of individual scholarly works. The article analysis was executed utilizing VOSviewer and CiteSpace, both prominent tools extensively applied across a multitude of academic evaluations, thereby providing indispensable perspectives for investigators within the pertinent discipline.

## 5 Conclusion

In this instance, we harnessed VOSviewer and CiteSpace for the bibliometric interrogation, assessing the contributions of nations, institutions, scholars, publications, and thematic concentrations. The ascending trajectory of annual scholarly output signals the burgeoning global scholarly interest in this domain. Beyond the robust collaborative nexus between the United States, Brazil, and China, there exists potential for enhancement in the cooperative endeavors amongst other nations. This analysis elucidates that the prevalent research foci are centered predominantly around the pathogenesis of *Cryptococcus*, the host immune response, immunological mechanisms, complications, and strategies for immunotherapy. In summation, this inquiry offers an invaluable compendium for academicians engaged in this sphere. Despite the complex pathogenesis of *Cryptococcus*, it is compelling to posit that the vigorous inquiry into the interplay between *Cryptococcus* and the host immune system will persist in bestowing considerable scholarly worth and propitious applications towards the identification of therapeutic targets.

## Data availability statement

The datasets presented in this study can be found in online repositories. The names of the repository/repositories and accession number(s) can be found below: https://www.webofscience.com/wos/woscc/summary/250318c0-253d-40f2-9755-506e08c996dc-c690f18e/relevance/1.

## Ethics statement

The manuscript presents research on animals that do not require ethical approval for their study.

## Author contributions

ST: Conceptualization, Data curation, Investigation, Software, Visualization, Writing – original draft, Formal analysis, Methodology, Project administration, Writing – review & editing. RH: Data curation, Methodology, Writing – original draft. XL: Formal analysis, Writing – original draft. HH: Data curation, Writing – original draft. YT: Formal analysis, Writing – original draft. TJ: Formal analysis, Writing – original draft. ZL: Formal analysis, Writing – original draft. XJL: Funding acquisition, Resources, Supervision, Writing – review & editing. YX: Funding acquisition, Resources, Writing – review & editing.
